# Characteristics of potential concussive events in elite hurling: a video-analysis study

**DOI:** 10.1007/s11845-023-03307-8

**Published:** 2023-02-17

**Authors:** Mario P Rotundo, Darek Sokol-Randell, Chris Bleakley, Paul Donnelly, Gregory Tierney

**Affiliations:** 1 Sport and Exercise Science Research Institute, Ulster University, Belfast, UK; 2 Department of Family Practice, University of British Columbia, Vancouver, Canada; 3 Department of Neurology, Memorial University of Newfoundland, St John’s, Canada

**Keywords:** Gaelic Athletic Association, Hurling, Sport-related concussion, Video analysis

## Abstract

**Background:**

High-impact sports such as hurling place participants at risk of sport-related concussion (SRC).

**Aims:**

This study will evaluate the characteristics of potential concussive events (PCEs) that occur in elite male hurling to acquire an understanding of how they occur.

**Methods:**

The authors recorded PCEs and their characteristics throughout two seasons of inter-county GAA competition using broadcast footage based on a previously validated protocol.

**Results:**

A total of 183 PCEs were identified over 82 inter-county matches (2.23 per match; 59.5 per 1000 h of exposure). PCEs that occurred in the 4th quarter were significantly more likely to result in signs of SRC. Players most often intended to receive/control the sliotar (36.4%, *n* = 64) prior to PCEs. The most frequently observed mechanism was shoulder-to-head (20.2%, *n* = 37). Impacts to the lateral aspect of the head were 2.7 times more likely to result in visible signs than impacts to anteroposterior regions.

**Conclusions:**

Players appear to be at a higher risk of SRC later in the match or when receiving the sliotar. Strikes to the lateral aspect of the head and those involving the shoulder appear to produce severe events. These findings provide initial guidance for the development of targeted player protection strategies.

## Introduction

SRC has recently been identified as a major public health concern in high-impact sports [1]. There are concerns surrounding the link between repeated mild traumatic brain injury and neurodegenerative disease such as CTE and Alzheimer’s disease [2–4]. While steps have been taken to combat this issue in various professional leagues, amateur and less globalized sporting associations may have fallen behind. The Gaelic Athletic Association (GAA) is the largest sporting body in Ireland and among the most highly regarded amateur sporting organizations in the world. The GAA presides over numerous sports and plays an influential role in preserving core values of Irish culture [5]. One of the most popular sports under the GAA’s purview is hurling, a game inscribed on UNESCO’s representative list of intangible cultural heritage of humanity [6].

Hurling is a game played between teams of 15 players and is often referred to as the fastest field sport on earth [7–9]. Players are required to wear helmets with full metal faceguards while using a wooden/composite hurling stick (hurley) to advance a small, hard leather ball (sliotar) down the pitch to score against an opposing team. The sliotar can be caught in the hand and carried for up to four steps, struck in the air, or struck on the ground with the hurley. To carry the sliotar for more than four steps, the player must balance or repeatedly bounce the sliotar on their hurley. This requires remarkable hand–eye coordination at high speeds as players advance the sliotar down the pitch and strike it either between the uprights or into the goal. While outright tackling is not permitted in sports such as rugby, shoulder-to-shoulder contact is encouraged, and high-velocity collisions are commonplace [10, 11]. Players also frequently use their hurleys to block and hook opponents to gain possession of the sliotar for their team.

We previously published a video-analysis study on the medical assessment of potential concussions in hurling. This study, using the same dataset, identified 183 PCEs over 82 matches, suggesting that head strikes are quite commonplace [12]. Surveys of GAA athletes have also suggested a high burden of SRC and insufficient management of these injuries [13]. Various steps have been taken to improve concussion assessment practices, such as the implementation of the GAA concussion management guidelines and, more recently, the announcement of a concussion interchange rule [14]. We have discussed this in previous papers on both Gaelic football and hurling [12, 15]. However, these measures do little to reduce the primary incidence or severity of concussion. Elucidating the characteristics and circumstances under which PCEs occur may allow us to establish targets for future research and player protection strategies [16]. This study intends to describe the characteristics of PCEs that occurred in national league and championship inter-county hurling over two playing seasons. The number of visible concussion signs present on video analysis will serve as a proxy for PCE severity and will be used to suggest which characteristics might be more likely to result in SRC. The results will then be interpreted to determine patterns and possible player protection strategies to be considered by the league.

## Methods

Video incident analysis has been demonstrated to be a valid method of analyzing situational factors, mechanisms, and signs of injury related to SRC. [17–20] We have previously used and reported a similar methodology for video analysis in Gaelic football and soccer. [16, 21] Consistent with prior work, a PCE is defined as any event in which a player is unable to resume play in a meaningful capacity within 5 s of direct and visible head contact [15, 21]. In other words, the player was either down on the ground, staggering, or otherwise disengaged from the play, therefore not participating purposefully or efficaciously in the gameplay around them. It is important to note that the term PCE is not synonymous with SRC; PCEs include a broad spectrum of head impacts that may or may not lead to a clinical diagnosis. Ambiguous events were excluded, such as those involving play-acting, questionable head contact, or minor contact where the blow could not possibly produce a concussive force. Play-acting was defined as a player appearing to take a dive or embellish an injury with the intention to gain a foul for his team. Although theoretically any contact to the head or body has the potential to cause concussion, we used our better judgment to exclude such contacts as finger grazes or events that did not produce any recoil, artifact, or reaction from the player whatsoever. To exclude a suspicious event, both reviewers had to agree upon the decision.

**Fig. 1:**
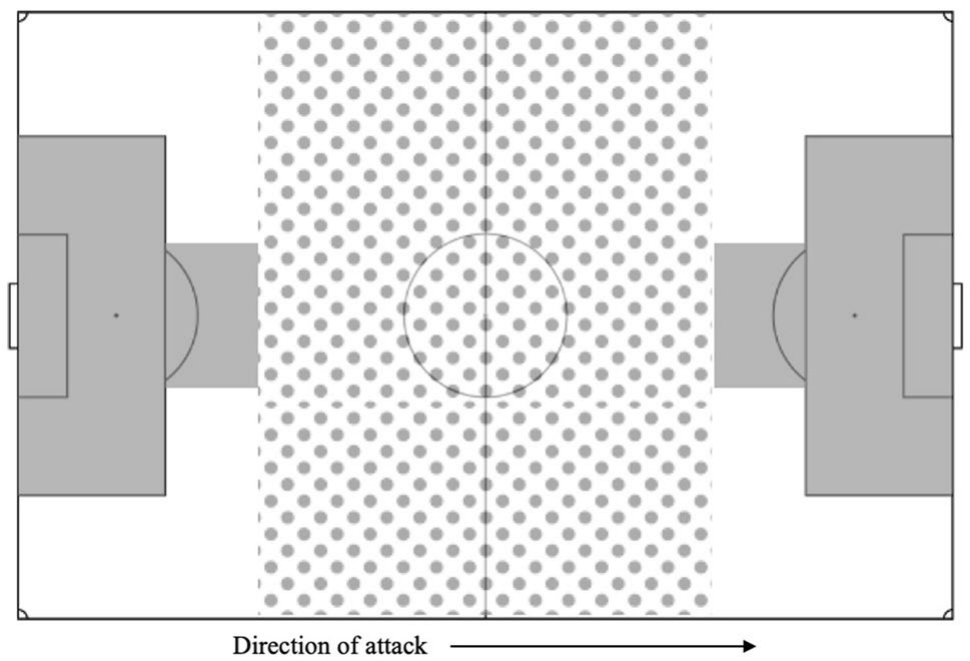
Recorded regions of the field where PCEs occurred including center (spotted), wings (white), and goal (gray)

**Fig. 2:**
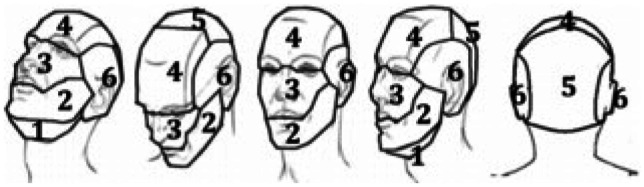
Recorded regions of head impact location involved in PCEs. These include (1) sub-mandibular, (2) mandibular, (3) maxillary, (4) frontal, (5) parieto-occipital, and (6) temporal

Reviewers were trained in video analysis at the injury prevention lab at St Michael’s Hospital in Toronto, Ontario, Canada, and have previously published PCE research in international soccer and Gaelic football. [12, 15, 16, 21, 22] Match footage was retrieved from the GAAGO online streaming service and was analyzed using QuickTime Player v10.5 (which enables frame-by-frame viewing). No patient data was utilized for this study, and all footage is public domain. Reviewers were permitted to rewatch and pan the footage at their discretion. In player-to-player contact, we defined “player 1” (P1) as the player who sustained head contact and “player 2” (P2) as an involved player who did not. If both players sustained head contact during a PCE, P1 was defined as the one who was assessed for a longer period, although both cases were still recorded as PCEs. Reviewers ascertained the time, context, intent of player, and area of the field where the PCE occurred (Fig. 1), as well as the mechanism and location of the impact (Fig. 2). Reviewers recorded whether the player appeared to anticipate the impending impact. The definition of anticipation was based on one previously established in rugby research, which classified the following as anticipated impacts: (i) player initiated the impact himself, (ii) player physically braces himself prior to impact, and (iii) player noted to visibly observe the impending impact [23]. Finally, consistent with our prior work and a recent international consensus statement, reviewers searched for the visible signs of concussion that are thought to be the most predictive of concussion has occurred. The presence of any one of these signs necessitates the immediate removal of an athlete from play, pending a professional assessment. [24] As mentioned in our introduction, these signs were used as a proxy for PCE severity in our analysis. When we refer to severe PCEs, we are referring to those that produced one or more visible signs of concussion and are therefore theoretically more likely to have resulted in true SRC. The complete analysis protocol is summarized in Table 1.

**Table 1:** Summary of peri-PCE characteristics analysis

***Pre-PCE***	***Mid-PCE***	***Post-PCE***
**Intent before injury (P1)** Action of P1 prior to injury	**PCE context** Context under which PCE occurred	**Card** Card awarded on play?
• Carry	• Hurley tackle	• Yellow
• Receive/control	• Aerial battle	• Red
• Challenge	• Body tackle	• Black
• Shoot	• Ground ball	
• Pass	• Other	**Foul awarded?**
• Malicious action		• Y/N
• Block		
• Other		
**Preceding event** Event occurring just prior to PCE	**Mechanism** Cause of strike leading to PCE	**Second hit** 2nd strike within 2 s of 1° impact
• Free	• Head	• Y/N
• Puck-out	• Hand/fist	
• 45	• Shoulder	
• Sideline cut	• Elbow	**Anticipation** Did player appear to expect impact?
• Short pass	• Arm	• Y/N
• Long pass	• Leg/hip	
• Interception	• Knee	
• Carry	• Foot/shin	
• Shot attempt	• Post	
• Bunch-up	• Ground	
	• Hurley	
	• Chest/back	
	• Other	
**Time quarter**	**Impact region** Region of head affected by impact	**Visible signs of concussion**
• 1/2/3/4	• Sub-mandibular	Lying motionless
	• Mandibular	Motor incoordination—ataxia
	• Maxillary	Impact seizure
	• Frontal	Tonic posturing
	• Occipital	No protective action—floppy
	• Parieto-temporal	Vacant look
	• Not visible	

Prior to analysis taking place, an inter-rater reliability test was performed. Each reviewer independently viewed five hurling matches and identified PCEs based on the criteria above, recording the exact minute of each incident. Next, each reviewer independently analyzed approximately 30 PCEs identified from exhibition and All-Ireland club matches (not included in data analysis) using the adapted PCE analysis spreadsheet. All discrepancies from the inter-rater reliability were discussed and clarified based on our comprehensive definitions. When a favorable result was achieved, reviewers independently identified PCEs throughout 59 and 52 matches of the 2018 and 2019 GAA National Hurling League inter-county seasons and championships, respectively. Each PCE was evaluated based on the parameters outlined above, and data were recorded for statistical analysis. Reviewers consulted each other and collaborated if any questions or difficulties arose during the analysis.

### Data analysis

Descriptive statistics were reported as means, counts, or frequencies and their associated percentages. A raw agreement and Cohen’s kappa coefficient were calculated for the inter-rater reliability test. Frequency distributions were used to examine patterns of PCEs based on time-quarter, context, anticipation, field region, mechanism, and impact location. A series of chi-squared tests were then performed to search for an association between these categorical variables and PCE severity (no signs vs. one or more visible signs of concussion). All expected cell counts must have been > 1, and no more than 20% of expected counts were < 5 [25]. If a contingency table violated this assumption, categories within a variable were combined logically and based on author consensus. For example, in our analysis of impact location, the mandibular and temporal regions were combined into lateral aspect of the head, and the frontal, maxillary, and occipital regions were combined into the anteroposterior regions.

When omnibus chi-square statistics were statistically significant (*p* < 0.05), we examined individual cell contributions using their residuals (difference between the observed (*O*) and expected values (*E*) for a cell), standardized to a *z* score [standardized residual = (*O* − *E*/√*E*)]. If applicable, we also compared cells of greatest interest using odd ratios (± 95% confidence intervals). We used log-linear tests to explore potentially important three-way interactions between sets of categoric variables and PCE severity.

## Results

### Inter-rater

Raw agreement was 100.0% for the identification of PCEs. A Cohen’s kappa was not calculated for identification of PCEs because the probability that both reviewers would record the same minute by chance is negligible. For the PCE analysis spreadsheet, raw agreement was 96.5% and Cohen’s kappa coefficient was 0.83 (95% CI 0.799 to 0.861) [26]. A Cohen’s kappa value greater than 0.8 is indicative of almost perfect agreement [27].

### Incidence

Throughout the 2018 and 2019 GAA inter-county hurling season, we identified a total of 176 incidents over 82 matches. Seven of these involved both P1 and P2 sustaining a PCE, resulting in 183 PCEs (2.2 per match; 59.5 per 1000 match hours of exposure).

### Game circumstances

We observed that 36 (19.7%) PCEs occurred in the first quarter of the match, 55 (30.1%) in the second quarter, 43 (23.5%) in the third quarter, and 49 (26.8%) in the fourth (Table 2). There was a significant association detected between the number of visible signs and match quarter, *χ*2 (3, *n* = 183) = 13.95, *p* = 0.0002. Posthoc testing confirmed the number of PCEs with at least 1 visible sign was significantly higher than expected in the 4th quarter of the match, based on a standardized cell residual of + 2.8. The greatest difference was found between the start vs the end of the match, with severe PCE being four and half times more likely to occur in the last quarter compared to the 1st quarter of play (OR 4.6 95% Cis 1.54 to 13.98).

**Table 2:** PCEs per quarter of match, by number of concussion signs observed (%)

**Time quarter**	**0**	**1 + **	**Total**
1	31 (86.1)	5 (13.9)	36 (19.7)
2	46 (83.6)	9 (16.4)	55 (30.1)
3	35 (81.4)	8 (18.6)	43 (23.5)
4	28 (57.1)	21 (42.6)	49 (26.8)
Total	140 (76.5)	43 (23.5)	**183 (100.0)**

### Impact location

**Fig. 3:**
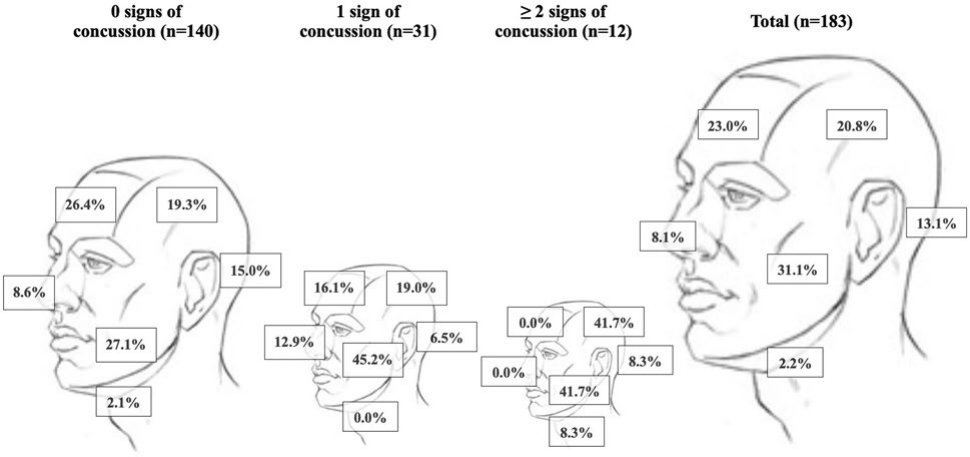
Percentage of PCEs affecting each region of the head, stratified by number of concussion signs produced

The mandibular region was the most impacted region, with 57 (31.2%) PCEs. Impacts to the frontal and temporal regions comprised 42 (23.0%) and 38 (20.8%) PCEs, respectively (Fig. 3). There was a significant association between impacted region and visible signs of concussion, *χ*2 (2, *n* = 183) = 7.19, *p* = 0.03. Post hoc tests found a higher-than-expected frequency of concussion signs associated with impacts to the lateral aspect of the head (mandibular/temporal) (standardized cell residual of + 1.6). Impacts to this region were 2.7 times more likely to result in severe PCEs compared to impacts involving the anteroposterior regions (frontal/maxillary/occipital) of the head (ORs 2.7 95% Cis 1.3–5.7, *p* = 0.0096).

### Mechanism

**Fig. 4:**
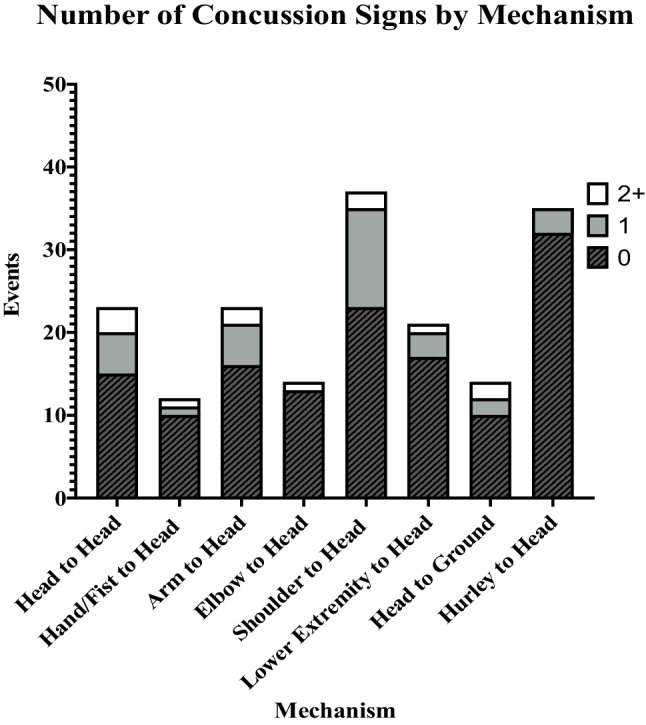
Number of concussive signs displayed, by mechanism of injury

When similar mechanisms were grouped together, the most common were shoulder to head (*n* = 37, 20.2%) and hurley to head (*n* = 35, 19.1%), followed by upper extremity (*n* = 49, 26.8%), head to head (*n* = 23, 12.6%), and lower extremity (*n* = 21, 11.5%). Shoulder-to-head impacts produced visible signs of concussion in 14 (37.8%) cases and head-to-head impacts in 8 (34.8%), compared to 3 (8.6%) in hurley-to-head impacts. This data is represented in Fig. 4.

The omnibus *χ*2 statistic was not significant between mechanism and visible signs, *χ*2 (5, *n* = 183) = 10.7, *p* = 0.06. However, post hoc tests found a higher-than-expected number of PCEs with visible signs due to shoulder-to-head impacts (*z* = 1.8). This mechanism was 6.5 times more likely to result in PCE with visible signs compared to hurley-to-head contact (OR 6.49, 95% Cis 1.67–25.22, *p* = 0.007). Head-to-head impacts were also more likely to produce visible signs compared to hurley to head (OR 5.68, 95% Cis 1.32–24.54, *p* = 0.019).

### Variable interactions

Taken together, 26 PCEs involved shoulder-to-mandible collisions (14.2%), 12 (46.2%) of which resulted in one or more visible signs of concussion. There was a clear association between impact location and PCE mechanism, *χ*2 (8, *n* = 183) = 39.79, *p* < 0.0001. Our post hoc tests determined that most impacts to lateral aspect of the head were caused by an opponent’s head or shoulder (43.6%, 43/95; *z* score = + 2.1), whereas impacts to the anteroposterior regions of the head were primarily caused by the hurley stick (34.1%, 28/82, *z* score + 3.1). We included both variables (impact location and mechanism) in a three-way log-linear analysis alongside injury severity, but the highest order interaction (impact location × mechanism × visible signs) was nonsignificant χ2 (8, = 3.36, *p* = 0.910).

### Player intent and pitch-related factors

For P1, the most common player intent preceding an incident was attempting to receive/control the sliotar (36.4%, *n* = 64), carrying the sliotar (31.3%, *n* = 55), and delivering a challenge (15.9%, *n* = 28). For P2, the most common intent was delivering a challenge (76.1%, *n* = 134). Detailed player intent data by pitch region and visible signs are displayed in Table 3.

**Table 3:** Intent prior to injury of both P1 and P2, by region and signs (%)

	***Pitch region***								***Signs***					
	***P1***				***P2***				***P1***			***P2***		
***Intent before INJ***	**Center**	**Goal**	**Wing**	***TOTAL (%)***	**Center**	**Goal**	**Wing**	***TOTAL***	***0***	***1* + **	***TOTAL***	***0***	***1* + **	***TOTAL***
**Carry**	19	29	7	**55 (31.3)**	3	3	3	**9 (5.1)**	44	11	**55 (31.3)**	0	0	**0**
**Receive/control**	28	19	17	**64 (36.4)**	11	5	2	**18 (10.2)**	49	15	**64 (36.4)**	2	0	**2 (28.6)**
**Challenge**	11	12	5	**28 (15.9)**	51	55	28	**134 (76.1)**	22	6	**28 (15.9)**	3	1	**4 (57.1)**
**Shoot**	3	1	0	**4 (2.3)**	1	2	0	**3 (1.7)**	2	2	**4 (2.3)**	0	0	**0**
**Pass**	7	4	5	**16 (9.1)**	1	1	0	**2 (1.1)**	12	4	**16 (9.1)**	0	0	**0**
**Malicious**	0	0	0	**0 (0.0)**	3	0	0	**3 (1.7)**	0	0	**0 (0.0)**	0	0	**0**
**Block**	1	2	0	**3 (1.7)**	0	0	0	**0 (0.0)**	1	2	**3 (1.7)**	0	0	**0**
**Other**	3	3	0	**6 (3.4)**	2	4	1	**7 (4.0)**	4	2	**6 (3.4)**	1	0	**1 (14.3)**
**Total (%)**	72 (40.9)	70 (39.8)	34 (19.3)	**176 (100.0)**	72 (40.9)	70 (39.8)	34 (19.3)	**176 (100.0)**	134 (73.2)	42 (23.0)	**176 (96.2)**	6 (3.3)	1 (0.55)	**7 (3.8)**

**Fig. 5:**
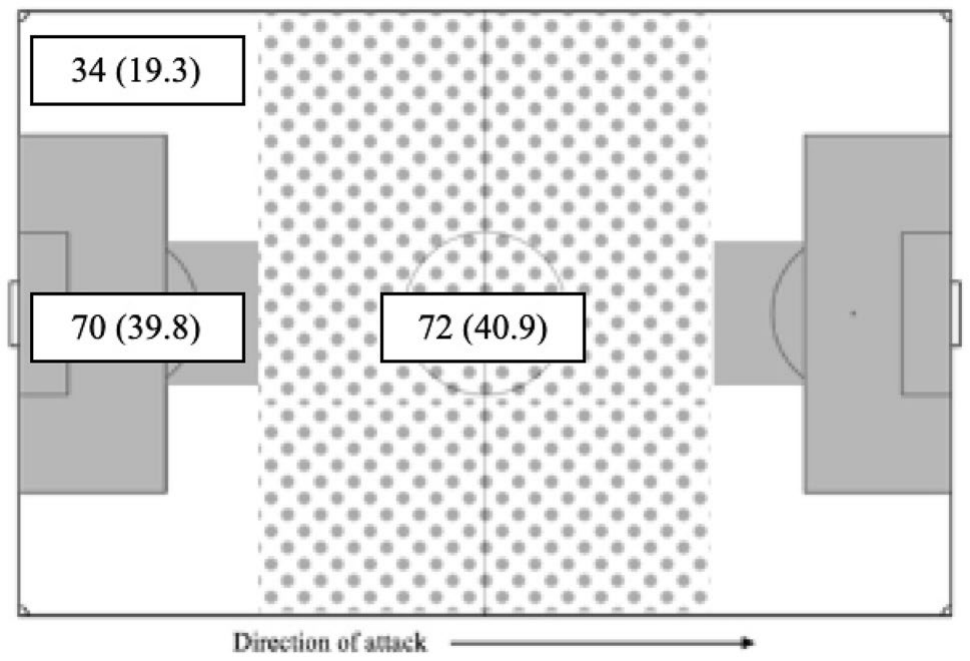
PCE incidents stratified by field region (%)

PCEs occurred with a similar frequency in the center and goal regions of the pitch. This data is displayed in Fig. 5. The most common contexts during which PCE occurred were body tackles (44.3%, *n* = 78), ground balls (24.4%, *n* = 43), and hurley tackles (18.2%, *n* = 32). PCE context data stratified by field region is displayed in Table 4. Seventy-seven (42.1%) players did not anticipate the impending impact that resulted in a PCE. There was a significant association detected between intent and anticipation (χ2 (6, *n* = 176) = 22.53, *p* < 0.001). Post hoc analysis revealed that when P1 intended to carry the ball, he was significantly more likely to anticipate a PCE. Unanticipated events were underrepresented during a carry (*z* = − 2.4), and players receiving/controlling the sliotar were 4 times less likely to anticipate an impending impact than in the event of a carry. (ORs 4.1 95% Cis 1.8–9.1, *p* = 0.0007).

**Table 4:** PCE context stratified by pitch region (%)

	***PCE context***					
***Pitch region***	**Hurley tackle**	**Aerial battle**	**Body tackle**	**Ground ball**	**Other**	**Total**
**Center**	19 (26.4)	9 (12.5)	28 (38.9)	14 (19.4)	2 (27.8)	**72 (40.9)**
**Goal**	9 (12.6)	4 (5.7)	38 (54.3)	14 (20.0)	5 (7.1)	**70 (39.8)**
**Wing**	4 (11.8)	2 (5.9)	12 (35.3)	15 (44.1)	1 (2.9)	**34 (19.3)**
**Total**	**32 (18.2)**	**15 (8.5)**	**78 (44.3)**	**43 (24.4)**	**8 (4.5)**	**176 (100.0)**

### Concussion signs

Of 183 PCEs, concussion signs were present in 43 (23.5%) cases. Two or more signs were present in 12 cases (6.6%) and three or more in 6 cases (3.3%). Lying motionless (*n* = 21) and blank/vacant look (*n* = 18) were the most common signs, followed by motor incoordination (*n* = 16).

## Discussion

Previous literature has reported 0.19–0.23 head injuries per 1000 h of hurling [28, 29], and various studies have indicated that head/neck injuries only account for 2.0–4.0% of injuries [28, 29]. Although a PCE does not necessarily indicate that a concussion has occurred, at 59.5 PCEs per 1000 match hours, our findings would suggest that there is a much higher incidence of concussion in hurling than is perhaps estimated.

PCEs were distributed relatively evenly throughout matches. However, 42.9% (*n* = 21) of PCEs occurring in the 4th quarter produced visible signs of concussion, representing a significantly higher-than-expected frequency than other quarters (Table 2). Our data suggests that severe PCEs were 4.5 times more likely to occur in the final quarter compared to the first. There are several possible reasons why this might be the case. First, it is possible that players are more prone to excessive aggression and impulsivity with prolonged physical exertion and as the game draws to a close [30], as has been demonstrated in ice hockey [31, 32]. Although our study only identified three instances of blatant malicious intent that led to PCE, all of these events occurred in the latter half of the match. Second, players may become more fatigued as the game progresses. GPS data demonstrates that mean total distance covered by hurlers is relatively consistent throughout a match. However, high-speed running distance and high-metabolic load distance (a figure combining HSR distance as well as acceleration/decelerations) tend to decrease quarter-by-quarter across all positions. Midfielders and half-forwards, who covered the most total distance and sprint distance in Q1, respectively, experienced the most profound reductions, suggesting that fatigue is a factor as the match progresses [33]. It is thought that physical resilience and tackle technique deteriorate with fatigue as players absorb numerous hits, leading to elevated susceptibility to head injury in the later stages of a match [34, 35]. This is consistent with studies in rugby and ice hockey, which detected a correlation between fatigue and elevated risk of concussion [36, 37]. Fatigued muscles are less able to absorb energy and, as a result, are likely more susceptible to injury.[38, 39] Although more research is needed to verify this, it is thought that the musculature of the neck plays an important role in neutralizing forces to the head, and neck strength has shown a significant inverse correlation with concussion risk [40, 41]. In rugby, tackling competency appears to decrease with increasing fatigue in sub-elite players [42] and the number of tackles a player is involved in has been positively correlated with markers of muscle damage [43, 44]. The combination of fatigued muscles and a commensurate decrease in tackle technique may explain why severe PCEs tended to occur later in the game.

PCE context is a parameter used to generate a general understanding of how PCEs occur. In most cases, defending players choose to either swing their hurley at an opponent to knock the sliotar from their possession (18.2%) or use a body tackle to knock their opponent off balance (44.3%). When organized by pitch region, body tackles were found to frequently occur in the goal region and hurley tackles in the center region of the pitch. Given the strategic importance of the goal region, it is reasonable to suggest that players are inclined to be more physical here, as has been similarly described in both Gaelic football and professional soccer [16, 21]. When we stratify context by intent, most PCEs (52.7%) occurring in the goal region involved P1 attempting to carry the sliotar, versus receive/control, which preceded only 29.7% (*n* = 19) PCEs in the goal region. Players will opt to carry the sliotar into the goal region if they are attempting to score on goal, rendering them more exposed to body tackles, while passes and puck-outs are most often received in the center and wing regions and tend to draw hurley tackles (Table 4).

Shoulder-to-head was the most common mechanism (20.2%, *n* = 37) and produced significantly more severe PCEs than expected (*z* = 1.8) (Fig. 5). While hurley-to-head was a comparably common mechanism (19.1%, *n* = 35), shoulder-to-head impacts were 6.5 times more likely to result in a severe PCE. To explain this phenomenon, we searched for an association between mechanism and impact location. Impacts to the lateral aspect of the head were most often caused by an opponent’s head/shoulder (43.6%, 43/95; *z* score = 2.1), whereas impacts to the anteroposterior regions of the head were primarily caused by the hurley stick (34.1%, 28/82, *z* score = 3.1). The lack of significant interaction between impact location, mechanism, and visible signs in a three-way log-linear analysis suggests that the strong association between impact location and PCE severity is not affected by underlying mechanism. For the purposes of player protection, it is helpful to know that shoulders tend to produce severe PCEs. This raises an important question: are shoulder-to-head impacts more dangerous due to the region of the head that they tend to affect?

**Fig. 6:**
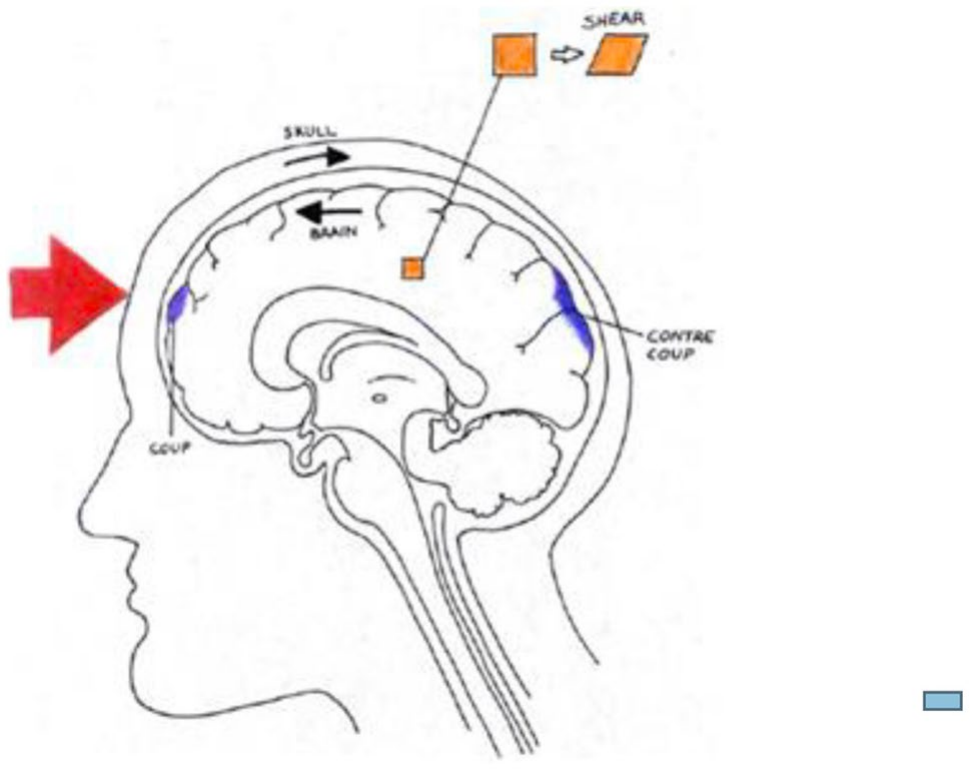
Forces on the brain during impact, including the coup-conter coup and shear mechanisms [51]

Similar to what we found in Gaelic football, it appears that when players are distracted, they are rendered susceptible to unanticipated “blind-side” collisions from approaching defenders [16]. We found that hurlers intending to receive or control the sliotar prior to when a PCE occurred were 4 times less likely to anticipate the impending collision compared to a player carrying the sliotar. Across multiple contact sports, unanticipated collisions have been recognized as a risk factor for SRC [16, 45–49]. When a player is looking away or unaware of an incoming tackle, important biomechanical implications for concussion risk may come into play. For one, the lateral aspect of the head may be rendered vulnerable. The brain is resistant to changes in volume but has limited resilience against changes in shape [50]. On impact, the brain tends to deform in shear (Fig. 6) [51], rendering it more sensitive to rotational loads [51, 52]. According to several studies, rotational loads produce more diffuse damage and relative brain motion than linear forces [53, 54], and impacts to the lateral aspect of the head appear to produce increased rotational forces and damage to the brain [55–57]. This effect may be compounded if the player does not anticipate the impending collision, as he cannot tense the musculature of the head and neck to absorb the impact [58–60]. This might explain why blows to the lateral aspect of the head were more likely to produce severe PCEs. It might also suggest why shoulder-to-head impacts, which were significantly more likely to affect the lateral aspect of the head, were significantly associated with severe PCEs. However, the exact causative mechanism of concussion remains quite contentious, and we can only speculate here as to why these results occurred.

### Implications

Hurling is an extremely fast-paced and physical sport that demands remarkable hand–eye coordination and attentiveness to the small and elusive sliotar, under the constant constraints of space and time. Based on the prevalence of PCEs and visible signs of concussion that we recorded over two seasons, it is reasonable to suggest that elite hurlers are at an elevated risk of concussion. This underscores the importance of identifying trends such as those discussed in this study to offer pointed suggestions on how to effectively reduce the incidence and severity of PCEs in hurling. It is worth noting the utility of video-analysis in this regard, and we continue to advocate for its usage for PCE surveillance and review, similar to what has been done in other sports [18, 61–63].

Given that PCEs tend to be more severe in the final quarter of the match, it is important that referees keep the game under control. The league might consider offering more substitutions to managers in the second half or allow a longer half-time to preserve tackle technique and resilience as the battle wears on. Adding neck strengthening protocols and emphasizing tackle technique in training may also be beneficial, as has been attempted with some success in rugby and American football, respectively [64, 65].

Our data suggests that player attention to incoming tackles is of paramount importance. PCEs tend to occur in situations where the player’s attention is diverted in some way, whether it be receiving or controlling a pass or attempting to corral a ground ball. These situations set the stage for blind-side strikes to the lateral aspect of the head, which as we explained above, can be extremely dangerous. We continue to advocate for severe penalties, suspensions, and fines for blind-side hits to the head, regardless of intent, to encourage players to be more deliberate and controlled with their tackles, especially when it may affect their team’s success. Variations of such interventions have been effectively integrated in the National Hockey League and National Football League regarding blind-side hits [47, 48, 66]. We have discussed this in more depth in our prior work [16].

Finally, stakeholder education with regard to concussion awareness, tackling technique, and protective strategies should be emphasized [67, 68]. Players should be taught from an early age that hits to the head, especially from the side on an unsuspecting opponent, can cause severe brain injury. Referees might approach high-risk situations with a heightened awareness and have a lower threshold for issuing fouls and cards if necessary. In training, coaches should emphasize “heads up” play and situational awareness, especially when receiving a pass in the center of the pitch or when carrying the sliotar into the face of the goal. The aptly named “heads up” style of play has been a fixture in ice hockey coaching for many years, which fortunately provides an existing framework that could likely be adapted to hurling [69–71].

## Limitations

The reviewers in this study were unable to control the movement of the camera, image quality, resolution of the athlete of interest, and angles available. This can influence the quality of the data collected. Some data points were difficult to assess in hurling compared to un-helmeted sports, such as anticipation and “vacant look.” However, most players removed their helmet after suffering a PCE, which mitigated this somewhat.

As explained in the methods, a PCE is not synonymous with SRC. PCEs include a broad spectrum of head impacts that may or may not lead to a clinical diagnosis. Despite a visible blow to the head, there is a possibility that players who subsequently disengaged from the play did so for a different reason, such as a coincident injury that we may have missed, leading us to overestimate the number of PCEs.

The hypothesis that PCEs were significantly more likely to occur in the 4th quarter of the match due to fatigue is limited by our failure to collect data on player substitutions. It is possible that some of the PCEs we noted to occur in the 4th quarter happened to players who entered the game later in the match. We were unable to find a reliable source for this data after the fact. Future research should take this into account.

Although a recent consensus study concluded that the six video signs of concussion used in this study are highly predictive of a concussion has occurred [24], reliance on video signs to predict a clinical diagnosis remains precarious. The identification of the signs of concussion relies on the reviewer’s interpretation of written definitions, introducing an element of subjectivity to this research. Furthermore, to our knowledge, the signs used in this study have not yet been validated based on clinical data, and not all SRCs present clinical signs on video. One study conducted on National Hockey League players showed that 53.0% of clinical concussions did not produce visual signs [72]. However, the signs in this study were only used as a proxy measurement for PCE severity and were only intended to suggest a higher probability of a concussion has occurred. For an event to qualify as a PCE, to begin with, the player had to suffer a direct, visible impact to the head and disengage from the play for 5 s or more. If we assume that approximately half of concussions present with visual signs, it is possible that this study drastically underestimated the number of PCEs that could be classified as severe. Unfortunately, we had no access to medical reports from the games, making it impossible for us to infer which PCEs were associated with clinical concussions. Statistically, the value of the three-way log-linear analysis between impact location, mechanism, and visible signs may have been impacted by the limited variety of mechanisms detected in our study. A more powerful sample would be needed to verify this statistic.

In the future, clinical diagnosis data would be beneficial for validating concussion signs and risk factors. Biomechanical analysis of PCEs, such as using instrumented mouthguards, would also offer valuable quantitative insight into risk factors and characteristics that lead to dangerous forces on the brain [52, 73].

## Conclusion

The current study intended to investigate the characteristics of PCEs in national league and championship inter-county hurling. High-risk characteristics were identified in relation to game time, player intent, mechanism, and impact location. For instance, impacts that occur later in the match and affect the lateral aspect of the head appear to produce more severe events. This provides us with a set of preliminary targets for intervention and future research. The league might consider implementing more severe penalties, especially for “blind-side” hits to the lateral aspect of the head, to encourage players to be more deliberate with their tackles. Compulsory concussion education offered to players and managers could raise awareness of high-risk situations and emphasize good tackling technique and heads-up play. Given the usefulness of video analysis demonstrated by our research, we continue to recommend that it be used for SRC surveillance, review, and future research. Inter-county hurling represents the highest caliber competition in the sport, and we are optimistic that targeted player protection strategies will meaningfully reduce the risk of concussion at all levels of the game without affecting pace and intensity. Ultimately, it is in everyone’s interest to keep talented young athletes healthy and active throughout their careers and beyond.

## References

[1] McCrory P, Meeuwisse W, Dvorak J et al (2017) Consensus statement on concussion in sport—the 5th international conference on concussion in sport held in Berlin, October 2016. Br J Sports Med bjsports-2017–097699. 10.1136/bjsports-2017-097699 10.1136/bjsports-2017-097699 28446457

[2] Ling H, Morris HR, Neal JW (2017). Mixed pathologies including chronic traumatic encephalopathy account for dementia in retired association football (soccer) players. Acta Neuropathol.

[3] Mackay DF, Russell ER, Stewart K et al (2019) Neurodegenerative disease mortality among former professional soccer players. N Engl J Med 1801–8. 10.1056/NEJMoa1908483 10.1056/NEJMoa1908483 PMC8747032 31633894

[4] Stewart WF, Kim N, Ifrah C (2018). Heading frequency is more strongly related to cognitive performance than unintentional head impacts in amateur soccer players. Front Neurol.

[5] Keller I, Wright A (2013) Amateurism in an age of professionalism: an empirical examination of an Irish sporting culture: the GAA. South West Open Research Deposit

[6] (2018) UNESCO List of Intangible Cultural Heritage - Hurling. https://ich.unesco.org/en/RL/hurling-01263. Accessed 7 Sep 2022

[7] (2015) Is hurling the fastest field sport in the world? In: The Irish Independent. https://www.independent.ie/storyplus/is-hurling-the-fastest-field-sport-in-the-world-31427432.html. Accessed 7 Sep 2022

[8] Harte D, Paterson A (2018). The fastest field sport in the world: a case report on 3-dimensional printed hurling gloves to help prevent injury. J Hand Ther.

[9] Kent D (2015). Eye safety in hurling: a few remaining blind spots?. Ir J Med Sci.

[10] Ly N (2015) The rules of hurling - explained!. https://www.youtube.com/watch?v=biFcgUB98ns

[11] Kavanagh B (2015) GAA biggest hits | Hurling & Football |

[12] Sokol-Randell D, Rotundo MP, Tierney G (2021). Video analysis of potential concussions in elite male hurling: are players being assessed according to league guidelines?. Ir J Med Sci.

[13] Sullivan L, Thomas AA, Molcho M (2017). An evaluation of Gaelic Athletic Association (GAA) athletes’ self-reported practice of playing while concussed, knowledge about and attitudes towards sports-related concussion. Int J Adolesc Med Health.

[14] Moran S (2021) GAA is expected to allow temporary replacement for head injuries. The Irish Times

[15] Sokol-Randell D, Rotundo MP, Tierney G (2021). Frequent but limited assessment of potentially concussed players in Gaelic football: an opportunity to learn from other sports. Ir J Med Sci.

[16] Sokol-Randell D, Rotundo MP, Tierney G et al (2021) Characteristics of potential concussive events in elite male gaelic football players: a descriptive video-analysis. J Sports Sci 1–9. 10.1080/02640414.2021.1896455 10.1080/02640414.2021.1896455 33722171

[17] Davis G, Makdissi M (2016). Use of video to facilitate sideline concussion diagnosis and management decision-making. J Sci Med Sport.

[18] Makdissi M, Davis G (2016). Using video analysis for concussion surveillance in Australian football. J Sci Med Sport.

[19] Gardner AJ, Kohler RMN, Levi CR, Iverson GL (2017) Usefulness of video review of possible concussions in National Youth Rugby League. Int J Sports Med 38. 10.1055/s-0042-116072 10.1055/s-0042-116072 27737484

[20] Davis GA, Makdissi M, Bloomfield P (2018). International study of video review of concussion in professional sports. Br J Sports Med.

[21] Armstrong N, Rotundo M, Aubrey J (2019). Characteristics of potential concussive events in three elite football tournaments. Inj Prev.

[22] Tarzi C, Aubrey J, Rotundo M (2020). Professional assessment of potential concussions in elite football tournaments.

[23] Gardner AJ, Howell DR, Iverson GL (2018). A video review of multiple concussion signs in National Rugby League match play. Sports Medicine - Open.

[24] Davis GA, Makdissi M, Bloomfield P (2019). International consensus definitions of video signs of concussion in professional sports. Br J Sports Med.

[25] Sharpe D (2015) Chi-Square Test is Statistically Significant: Now What? University of Massachusetts Amherst. 10.7275/TBFA-X148

[26] McHugh ML (2012) Interrater reliability: the kappa statistic. Biochemia Medica 276–82. 10.11613/BM.2012.031 PMC3900052 23092060

[27] Landis JR, Koch GG (1977). The measurement of observer agreement for categorical data. Biometrics.

[28] Blake C, John M, Conor G, O’Malley E (2014). Injury to the head region in elite male Gaelic football and hurling: 2007–2012. Br J Sports Med.

[29] Blake C, O’Malley E, Gissane C, Murphy JC (2014) Epidemiology of injuries in hurling: a prospective study 2007–2011. BMJ Open 4:e005059. 10.1136/bmjopen-2014-005059 10.1136/bmjopen-2014-005059 PMC4067887 24948748

[30] Schmit C, Brisswalter J (2020). Executive functioning during prolonged exercise: a fatigue-based neurocognitive perspective. Int Rev Sport Exerc Psychol.

[31] Kirker B, Tenenbaum G, Mattson J (2000). An investigation of the dynamics of aggression: direct observations in ice hockey and basketball. Res Q Exerc Sport.

[32] Widmeyer NW, McGuire EJ (1997). Frequency of competition and aggression in professional ice hockey. Int J Sport Psychol.

[33] Young D, Kilty J, Hennessy L, Coratella G (2020). The running performance decrement in elite hurling. Appl Sci.

[34] Tierney GJ, Denvir K, Farrell G, Simms CK (2018). Does player time-in-game affect tackle technique in elite level rugby union?. J Sci Med Sport.

[35] Tierney GJ, Lawler J, Denvir K (2016). Risks associated with significant head impact events in elite rugby union. Brain Inj.

[36] Stevens ST, Lassonde M, de Beaumont L, Keenan JP (2008). In-game fatigue influences concussions in national hockey league players. Res Sports Med.

[37] Hollis SJ, Stevenson MR, McIntosh AS (2009). Incidence, risk, and protective factors of mild traumatic brain injury in a cohort of Australian nonprofessional male rugby players. Am J Sports Med.

[38] Landry M (2014) Brukner & Khan’s clinical sports medicineBrukner & Khan’s clinical sports medicine, 4th ed. Brukner Peter Khan Karim Sydney: McGraw-Hill Australia; 2012 ISBN-13 978–0–07099–813–1 1268 p., illus. CAD$167.95. Physiotherapy Canada 66:109–110. 10.3138/ptc.66.1.rev2

[39] Mair SD, Seaber AV, Glisson RR, Garrett WE (1996). The role of fatigue in susceptibility to acute muscle strain injury. Am J Sports Med.

[40] Collins CL, Fletcher EN, Fields SK (2014). Neck strength: a protective factor reducing risk for concussion in high school sports. J Prim Prev.

[41] Hanson E, Stracciolini A, Mannix R, Meehan WP (2014). Management and prevention of sport-related concussion. Clin Pediatr.

[42] Gabbett TJ (2008). Influence of fatigue on tackling technique in rugby league players. J Strength Cond Res.

[43] Takarada Y (2003). Evaluation of muscle damage after a rugby match with special reference to tackle plays. Br J Sports Med.

[44] Smart DJ, Gill ND, Beaven CM (2008). The relationship between changes in interstitial creatine kinase and game-related impacts in rugby union. Br J Sports Med.

[45] Guskiewicz KM, Mihalik JP (2006). The biomechanics and pathomechanics of sport-related concussion. Foundations of Sport-Related Brain Injuries.

[46] National Hockey League (2010) Rule prohibiting lateral, back-pressure or blind-side hit to head will take effect

[47] Donaldson L, Asbridge M, Cusimano MD (2013). Bodychecking rules and concussion in elite hockey. PLoS ONE.

[48] Shaw S, Halpin T, Stubberman M (2019) 2019 NCAA football rules and interpretations

[49] Mihalik JP, Blackburn JT, Greenwald RM (2010). Collision type and player anticipation affect head impact severity among youth ice hockey players. Pediatrics.

[50] Bradshaw, D, Morfey C (2001) Pressure and shear response in brain injury models. In: Proceedings of the 17th international technical conference on the enhanced safety of vehicles. Amsterdam, The Netherlands

[51] Kleiven S (2013). Why most traumatic brain injuries are not caused by linear acceleration but skull fractures are. Front Bioeng Biotechnol.

[52] Tierney G (2021) Concussion biomechanics, head acceleration exposure and brain injury criteria in sport: a review. Sports Biomech 1–29. 10.1080/14763141.2021.2016929 10.1080/14763141.2021.2016929 34939531

[53] Adams JH, Graham DI, Murray LS, Scott G (1982). Diffuse axonal injury due to nonmissile head injury in humans: an analysis of 45 cases. Ann Neurol.

[54] Unterharnscheidt F, Higgins LS (1969). Traumatic lesions of brain and spinal cord due to nondeforming angular acceleration of the head. Tex Rep Biol Med.

[55] Yoganandan N, Gennarelli TA, Zhang J (2009). Association of contact loading in diffuse axonal injuries from motor vehicle crashes. J Trauma.

[56] Zhang J, Yoganandan N, Pintar FA (2009). Dynamic biomechanics of the human head in lateral impacts. Ann Adv Automot Med.

[57] Zhang L, Yang KH, King AI (2001). Comparison of brain responses between frontal and lateral impacts by finite element modeling. J Neurotrauma.

[58] Bauer JA, Thomas TS, Cauraugh JH (2001). Impact forces and neck muscle activity in heading by collegiate female soccer players. J Sports Sci.

[59] King D (2018). Head impact biomechanics: Comparison between sports and genders. J Sci Med Sport.

[60] Rowson S, Duma SM, Beckwith JG (2012). Rotational head kinematics in football impacts: an injury risk function for concussion. Ann Biomed Eng.

[61] National football league operations (2020) ATC SPOTTERS

[62] Mack C, Myers E, Barnes R (2019). Engaging athletic trainers in concussion detection: overview of the national football league ATC spotter program, 2011–2017. J Athl Train.

[63] Comper P, Echemendia R, Armstrong D et al (2016) NHL concussion evaluation and management protocol - 2016/2017 Season. 1–7

[64] Geary K, Green BS, Delahunt E (2014). Effects of neck strength training on isometric neck strength in rugby union players. Clin J Sport Med.

[65] Kerr ZY, Yeargin SW, Valovich McLeod TC (2015). Comprehensive coach education reduces head impact exposure in American Youth Football. Orthop J Sports Med.

[66] Goodell R (2019) Official playing rules of the NFL. 90

[67] Tierney GJ, Simms CK (2017). Concussion in rugby union and the role of biomechanics. Res Medica.

[68] Cross MJ, Tucker R, Raftery M (2019). Tackling concussion in professional rugby union: a case-control study of tackle-based risk factors and recommendations for primary prevention. Br J Sports Med.

[69] Ashare AB (2020) Heads up, don’t duck program for decreasing the risk for cervical spine injury. In: Smith TA, Ashare AB (eds) Safety in Ice Hockey: 6th Volume. ASTM International, 100 Barr Harbor Drive, PO Box C700, West Conshohocken, PA 19428–2959, pp 108–116

[70] Ben MacAskill (2016) Heads up hockey: training upward gaze while stick-handling. Dalhousie University

[71] Ashare AB (2020) Heads Up, Don’t Duck Program for Decreasing the Risk for Cervical Spine Injury. In: Smith TA, Ashare AB (eds) Safety in Ice Hockey: 6th Volume. ASTM International, 100 Barr Harbor Drive, PO Box C700, West Conshohocken, PA 19428-2959, pp 108–116

[72] Echemendia RJ, Bruce JM, Meeuwisse W (2018). Can visible signs predict concussion diagnosis in the National Hockey League?. Br J Sports Med.

[73] Tooby J, Weaving D, Al-Dawoud M, Tierney G (2022). Quantification of head acceleration events in rugby league: an instrumented mouthguard and video analysis pilot study. Sensors.

